# Does the application of Opsite^⋄^ Flexigrid^⋄^ occlude the oxygen saturation readings in healthy individuals using the moorVMS-OXY machine?

**DOI:** 10.1186/s13047-020-00391-2

**Published:** 2020-05-12

**Authors:** A. Beaumont, L. McSorley, M. Matthews, K. Mooneesawmy, L. Little, J. R. Forss

**Affiliations:** Centre for Regenerative Medicine and Devices, University of Brighton, 49 Darley Road, Eastbourne, BN20 7UR UK

**Keywords:** Oxygen saturation percentages, Opsite Flexigrid, First metatarsophalangeal joint, Skin stripping, MoorVMS-OXY, Cross-infection

## Abstract

**Background:**

A proportion of people who have been diagnosed with peripheral arterial disease and diabetes mellitus will be susceptible to chronic wounds. Oxygen is vital for wound healing, so oxygen measurements should to be taken as predictive values for wound healing in patients. When measuring oxygen at the wound bed, there is potentially a risk of cross-infection if no protective barrier is used; and skin stripping if an adhesive barrier is used on the wound bed. This cross sectional within subject repeated measures pilot study, aims to determine if the application of opsite film, as an infection control measure, in one or two layers, impacts on tissue oxygenation readings obtained when using the MoorVMS-OXY.

**Methods:**

Mean oxygen saturation percentages were measured from 29 limbs of 18 healthy participants. Oxygen saturation was measured for 20 s and analysed at the first metatarsophalangeal joint using no film, one and two layers using the MoorVMS-OXY. A one-way repeated ANOVA with a Bonferroni post hoc test was performed to test for statistically significant differences between the values of the three parameters and multiple pairwise comparisons was completed.

**Results:**

Amongst the three layers, there was a statistically significant difference in oxygen saturation between the two layers of Opsite Flexigrid and none; and also between the two layers of Flexigrid and single layer (*p* < 0.05). It was also established that there was no statistically significant difference between the single layer of Opsite Flexigrid and no Flexigrid layer (*p* > 0.05).

**Conclusions:**

The results imply that one layer of Opsite Flexigrid is a suitable protective barrier to use when establishing capillary bed oxygen perfusion with the MoorVMS-OXY. However, the application of two Opsite Flexigrid layers, to prevent skin stripping, decreases the recorded values of oxygen saturation percentages significantly, therefore providing inaccurate results. Indicating that a double layer cannot be used over ulceration sites if measuring oxygen levels at the wound bed.

## Background

A proportion of people who are diagnosed with peripheral arterial disease (PAD) and diabetes mellitus may develop a chronic wound [1]. A chronic wound is a break in the skin which has failed to go through each stage of wound healing (hemostasis, inflammation, proliferation and maturation) in the correct time frame [2]. This can be longer than 6 weeks or reoccurs frequently [3, 4].

Oxygen is a vital component for wound healing and a lack of oxygen can impact every stage of the healing process [2]. During wound healing, there is increased demand for energy which is provided when oxygen is readily available [5]. Energy is a fundamental requirement in wound healing due to the increased need to aid reparative processes such as, bacterial defences to prevent infection, cell proliferation and synthesis of collagen [6].

Oxygen levels need to be assessed as a decrease of oxygen in the body is associated with the development of ischemia, PAD and a reduced healing rates [2]. There are different methods for establishing arterial limb perfusion. In routine podiatric practice, this is primarily achieved via the use of a hand held Doppler, which focus’ on arterial perfusion of the foot by assessing the dorsalis pedis and the posterior tibial arteries. A study by Ladner et al., [7] showed that transcutaneous tissue oxygen tension measurement (TcPO_2_) is also a suitable clinical screening tool for estimating the risk of foot ulcer non-healing in patients with diabetes and absent palpable pedal pulses. When considering the structures assessed using this device, its focus is on arteriole and capillary perfusion, which can provide vital information in relation to the perfusion of the tissues in the region of the wound. Limitations of the probes of this device are that they heat the tissues underneath, they are larger, more numerous and placed only around the wound margins. This does give useful information regarding the wound periphery but does not give actual wound bed perfusion values. The MoorVMS-OXY does provide the means of evaluating capillary oxygen saturation at the wound bed by utilizing white light reflectance spectroscopy [8]. There is only one, smaller probe to apply, which does not need to heat the tissues, therefore there is no delay prior to taking tissue measurements. The use of this device would enable clinicians to identify individuals who may have normal artery and arteriole flow but have capillary bed perfusion problems, which currently are not being assessed. These two devices mentioned are not commonly used in podiatry practice, but could provide valuable information if utilized more frequently [6, 9]. Clinicians may be concerned with applying a probe to the wound bed itself due to infection control reasons, concerns with cleaning the probe after use (as they are not disposable), causing skin stripping to the peri-wound skin on removal of a protective barrier film, if used, as well as uncertainty as to whether the protective barrier film used could affect the reliability of readings obtained from the device.

Measuring oxygen saturation percentages (SO_2_%) at a wound bed could place the patient at risk of infection if performed incorrectly. All healthcare professionals are involved with infection prevention and control and must adhere to the quality standards to protect those who are vulnerable [10]. To measure ulcers, infection prevention methods must be in place. There are sterile transparent film dressings, which allow the exchange of oxygen and water vapour [11, 12], which could be used to allow devices to measure SO_2_% whilst minimizing the risk of cross-infection and allowing faster probe cleaning, post use [13, 14]. However, these dressings adhere to the skin, if used in a single layer, and there is the possibility that this could leave the patient susceptible to skin stripping on it’s removal [14–17]. Skin stripping of the peri-wound can lead to an increase in the wound size and additionally delay the healing process [14–17]. If these dressings were used in a double layer, the skin stripping element would be removed but the thickness of the dressing may have a detrimental effect on the machine readings.

This study aims to answer this question, does the application of opsite film, as an infection control measure, in one or two layers, impact on tissue oxygenation readings obtained when using the MoorVMS-OXY? This would then allow clinicians to measure the oxygen saturation at the wound bed without the patient being at risk of cross-infection or skin stripping [14–17], while maintaining confidence in the values that are obtained.

## Methods

### Study design

In this quantitative within-subject repeated measure pilot study, the same participants limb was used for the three different experimental conditions; no Opsite Flexigrid, single Opsite Flexigrid and double Opsite Flexigrid. SO_2_% were taken from these experimental conditions measured on the planter surface of the 1st metatarsophalangeal joint (MTPJ) using the MoorVMS-OXY.

### Participants

For this study 27 participants volunteered, Twenty participants attended for the first appointment where written consent and eligibility screening was performed. All participants met the inclusion criteria, however, only eighteen participants returned for the data collection appointment. The participants did not have any history of anemia, PAD, previous myocardial infarction, heart attack, sickle cell disease, leukemia, carbon monoxide poisoning, and monophasic pulses. All Participants had a Fitzpatrick score of 1 or 2, alongside biphasic or triphasic pulses (using a Doppler Ultrasound) and good skin integrity. The Fitzpatrick skin classification system is a tool which can be used to classify the amount of melanin pigment in the skin along with how the skin reacts following exposure to sunlight. The MoorVMS OXY has been validated for Fitzpatrick skin types 1 and 2, and is reported to not be as effective in the other skin classification types due to the melanin reflecting the white light of the device [18]. In addition, the following were excluded: under 18 years old, being a present smoker, pregnant, a high alcohol intake (over 14 units a week), an allergy to Opsite Flexigrid, latex or Mefix tape and a skin type of 3 to 6 on the Fitzpatrick Scale [18].

Of the original twenty consented participants, eighteen participants returned for the second step of the project. Of these eighteen recruited (36 limbs), the data collected from 7 limbs was used to optimize the data collection method and ensure efficient use of the MoorVMS-OXY and thus was excluded from the final data set, leaving the data from 29 participant limbs being used for the data analysis. The mean Participant age was 27.6 years old (range: 20–48).

All participants signed a written consent in accordance with the ethics committee on human subjects as required by the university before participation. The study protocol was approved by the School Research Ethics and Governance Panel, University of Brighton on the 21st June 2017.

### Instrumentation and equipment protocol

MoorVMS-OXY (moorinstruments) is a white-light reflectance spectroscopy device, using wave lengths ranging from 500 nm–650 nm [8, 19]. For an overall tissue SO_2_%, back-scattered light reflects the concentration of haemoglobin in the red blood cells [8, 19]. This measures the haemoglobin SO_2_% in the capillaries and veins of microcirculation in the tissue, providing evidence of changes in the microvascular blood supply and tissue oxygen consumption [8, 9, 19].

The probe (PH1-V2) used measures to a tissue depth of 1-2 mm of the upper tissue [8]. SO_2_% were recorded on the 1st MTPJ of each participant using the MoorVMS-OXY and MoorVMS-PC program to collect anonymized participant data. The measurements, at this site, were performed in triplicate for each participant with the following conditions, no Opsite Flexigrid, Opsite Flexigrid and double Opsite Flexigrid. The probe cleansing, application and collection protocol was obtained following guidance from the user manual [19]. For the probe to be attached onto the 1st MTPJ, the probe was attached to the probe holder and moorPAD adhesive discs were attached to the probe to adhere onto the 1st MTPJ. A calibration check was made before each measurement by following the manufacturer’s guidelines. The detected signal was processed and displayed as ‘calibration successful’. The measureable range of SO_2_ is 0–99%, however a ‘normal’ value of SO_2_ in the leg or arm ranges from 20 to 50%, and the lesser digits 50–80% [8]. Data was collected continuously over a 30 s period for each test condition, whereby a 20 s region of interest for each layer was selected for analysis (5 s off the start and end of each 30 s period). The MoorVMS-OXY performs a reading every 0.025 s, therefore, during the 20 s data collection period, 800 individual data capture points were collected. This data was used to calculate the means, standard deviations (SD) and SD errors were calculated for the three test environments on each participant. All data collected is expressed as mean **±** SD.

Opsite Flexigrid is a sterile, transparent, semi-permeable film wound dressing made of a polyurethane membrane with an acrylic adhesive layer (Smith & Nephew UK, Cutifilm) [20]. As it is semipermeable, it means it is permeable to water vapour and oxygen, however it is impermeable to micro-organisms [20, 21]. A single Opsite Flexigrid was used because Opsite Flexigrid is an effective barrier to external contamination, reducing the risks of cross-infection. It allows excess exudate to evaporate while providing a moist environment at the surface of the wound to ensure an optimum wound healing environment [20, 21]. However, there is an acrylic adhesive layer, which is reasonable for patients who have no skin integrity issues and are not at risk to infection [20, 21].], using this on a patient with an ulcer could lead to skin stripping [17]. Opsite Flexigrid has a tendency for skin stripping to occur on a new epidermis from recently re-epithelized areas of the wound [11, 22]. So, to prevent skin stripping occurring a double Opsite Flexigrid was made by folding one Opsite Flexigrid into two (adhesive sides adhered together) where Mefix held the double Opsite Flexigrid in place. For the single Opsite Flexigrid, the dressing (size 6 × 7 centimetres) was cut in half.

To ensure accuracy of the data collected, each researcher did the same task throughout the project. The data collection ran for a period of 4 weeks. There were two appointments given, the first appointment was to collect participant information for inclusion, exclusion criteria, and gaining informed, written consent. All data collected was coded for participant anonymity.

If the participant passed the inclusion, exclusion criteria, a second appointment was booked. However, at least 24 h was given before that appointment to allow participants to opt out if they wanted to. On the second appointment, the data was collected on both limbs of each participant.

For data collecting, firstly, the participants had to acclimatise in the treatment room, the observed room temperature was observed to be 21-24 °C for 15 min [23] in a resting upright sitting position, with feet elevated.

Prior to data collection, the foot was wiped using a standard clinical procedure (patient Carell wipes) before setting up the device and following Moor VMS protocol. The probe was cleaned before and after use following manufacturer recommendations [19]. To ensure accuracy of the data collected, a sterile surgical marker was used, where an area on the 1st MTPJ was outlined, to enable the same position to be measured for each layer. The 1st MTPJ was measured as previous research has suggested that it is the most common weight-bearing ulceration site [24, 25].

The MoorVMS-OXY was switched on, calibrated and then the probe was attached onto the outlined area. For no layer of Opsite Flexigrid, the data was collected for 30 s, then the device was paused. Whilst it was paused, the probe was removed, then a sterile single Opsite Flexigrid was applied over the same area, recorded data for another 30 s, then paused again. The process was again repeated for the sterile double Opsite Flexigrid (making sure there were no air bubbles) held using Mefix.

The data was collected using the moorVMS-PC program alongside the MoorVMS-OXY, then transferred onto Excel to plot the data.

### Statistical analysis

Statistical analysis was conducted using IBM SPSS Statistics Desktop, version 24.0, Mac OS. All data was tested for normality using the Shapiro-Wilk test. Means and SD was calculated for each variable. A one-way repeated Analysis of Variance (ANOVA) was used to test for significant differences in SO_2_% across the within-subjects factor (no Opsite Flexigrid, single Opsite Flexigrid, double Opsite Flexigrid). A Bonferroni post hoc test was used for multiple pairwise comparisons between the three variables whilst also providing the statistical significance level for each pairwise comparison. The level of significance was set at *p* < 0.05.

## Results

**Fig. 1:**
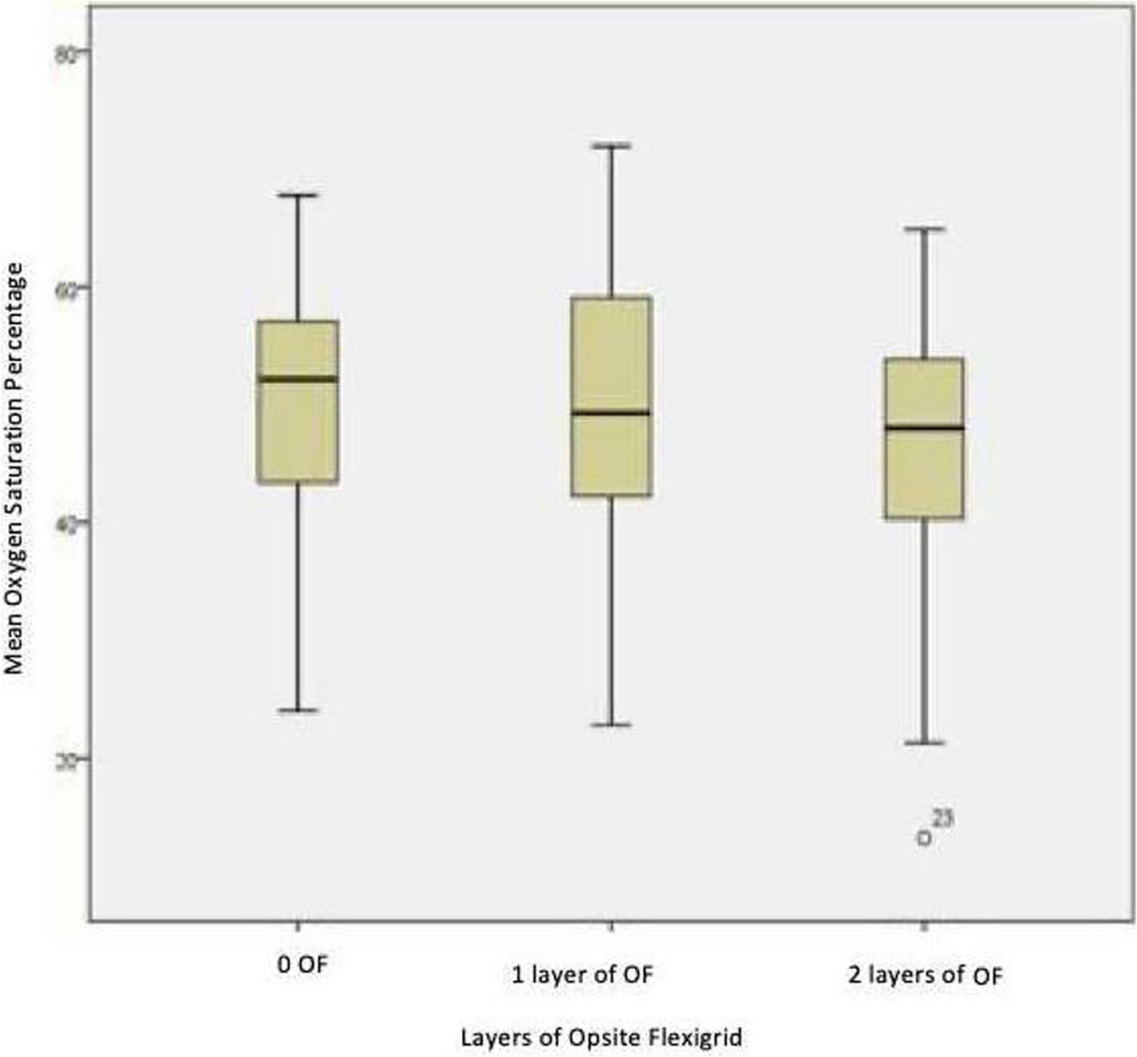
Oxygen saturation percentages compared to layers of Opsite Flexigrid

The eighteen participants included undertook the protocol without complications and no participants withdrew from the study. The data was obtained from 29 of the available 36 participant limbs. For the purposes of calculating the results n = the number of participant limbs included in the study will be used, thus *n* = 29. The data was normally distributed, as assessed by box plotting, see Fig. 1, and performing a Shapiro-Wilk test on the results, *p*-value is greater than 0.05 (no Opsite Flexigrid: *p* = 0.267, single Opsite Flexigrid: *p* = 0.331, double Opsite Flexigrid: *p* = 0.148) respectively. Mauchly’s test of sphericity showed that the assumption of sphericity had not been violated.

Descriptive Statistics showed that the highest measure of SO_2_% occurred at the 1st MTPJ with no Opsite Flexigrid (mean: 49.1241, SD: 11.59990), followed by a slight SO_2_% decrease with a single layer of Opsite Flexigrid (mean: 48.7241, SD: 12.77298). However, an even further SO_2_% decrease with a double Opsite Flexigrid at the 1st MTPJ (mean: 45.2069, SD: 12.46208).

Figure 1 shows the mean SO_2_% of each participant from the samples across the independent variables. This shows that as the OF layers increase the SO_2_% decrease. The SO_2_% ranges from layer to layer; no Opsite Flexigrid ranges from 24.0–67.8%, single Opsite Flexigrid ranges from 23.6–72.0% and double Opsite Flexigrid ranges from 13.2–64.9%.

After performing the one way repeated ANOVA, it was shown that SO_2_% was statistically significant at the different layers; F (2,56) = 4.501, *p* < 0.05, *p* = 0.015. Pairwise comparisons further showed the mean differences in SO_2_% between each of the layers and whether it is statistically significantly different or not. See Table 1. A single layer of Opsite Flexigrid compared to no Opsite Flexigrid has a decreased mean difference of 0.400, however was not statistically significant different because *p* > 0.05, *p* = 1.000 (95% confidence interval, − 4.584 to 3.784). The mean differences for a double layer of Opsite Flexigrid versus no Opsite Flexigrid decreased by 3.917, and double layer of Opsite Flexigrid versus a single layer of Opsite Flexigrid decreased by 3.517. This shows that mean values for double Opsite Flexigrid versus no Opsite Flexigrid (*p* = 0.037, 95% confidence interval, − 7.640 to − 0.195), and double Opsite Flexigrid versus single Opsite Flexigrid (*p* = 0.016, 95% confidence interval, − 6.484 to − 0.551) have a statistically significant difference (*p* < 0.05).

**Table 1:** Pairwise Comparisons

Pairwise Comparisons			Measure: SO2		
Layers	Layers	Mean Difference	Sig. ^**b**^	95% Confidence Interval for Difference ^**b**^	
				Lower Bound	Upper Bound
0 Layers	1 Layer	.400	1.000	−3.784	4.584
	2 Layers	3.917^a^	.037	.195	7.640
1 Layer	0 Layers	−.400	1.000	−4.584	3.784
	2 Layers	3.517^a^	.016	.551	6.484
2 Layers	0 Layers	−3.917^a^	.037	−7.640	−.195
	1 Layer	−3.517^a^	0.16	−6.484	−.551

Based on estimated marginal means

^a^ The mean difference is significant at the 0.05 level

^b^ Adjustment for multiple comparisons: Bonferroni

### Estimated marginal means of oxygen saturation

**Fig. 2:**
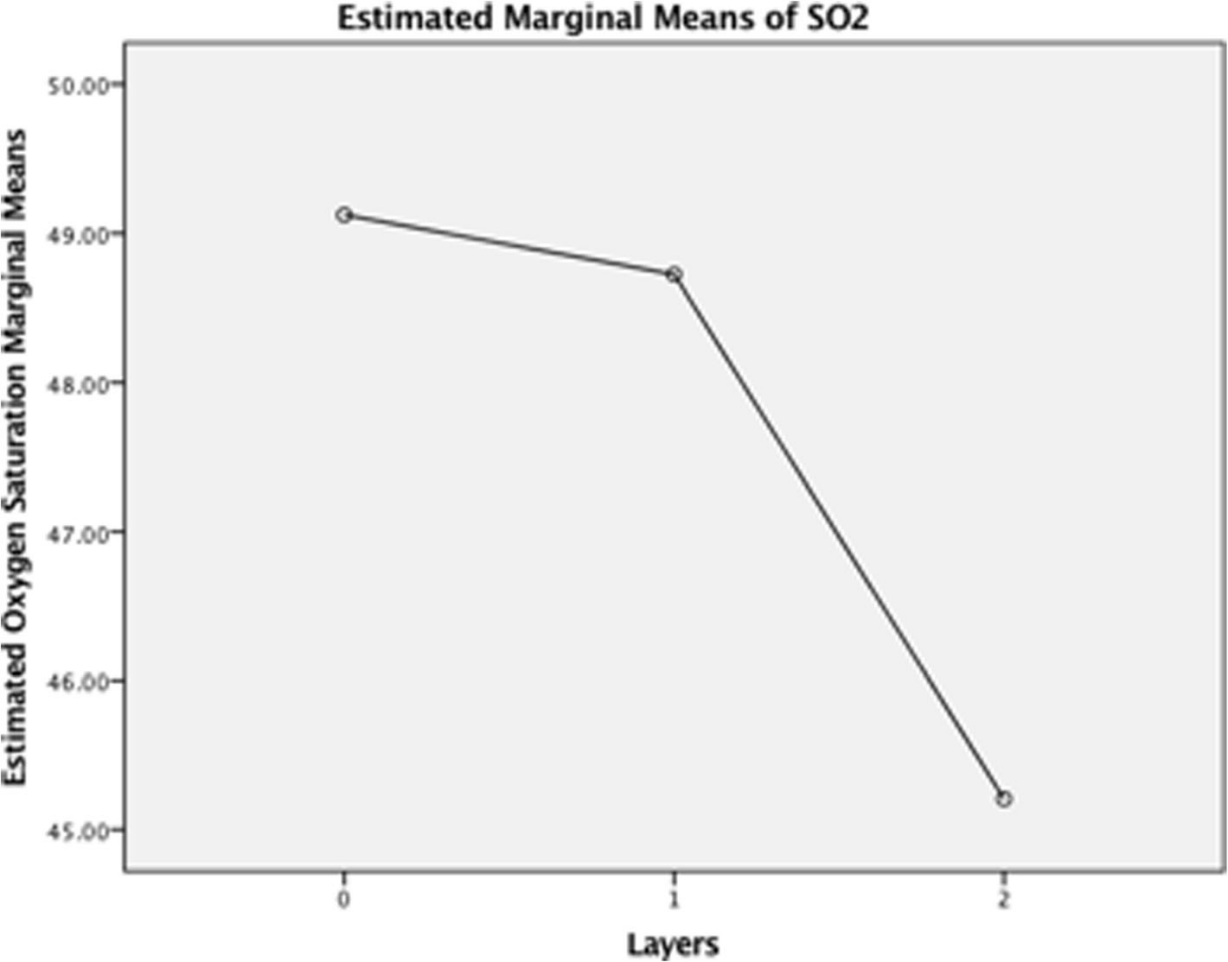
Estimated Marginal Means of Oxygen Saturation

There is a general negative correlation of mean SO_2_%, illustrated in Fig. 2. There is a high estimated SO_2_% marginal mean for no Opsite Flexigrid, with a gradual decrease for a single layer of Opsite Flexigrid, then a rapid decrease for a double layer of Opsite Flexigrid.

Therefore, the evidence shows that Opsite Flexigrid does affect SO_2_% compared to when no Opsite Flexigrid is used. The findings specifically suggest that a double Opsite Flexigrid greatly affects SO_2_%, more so than a single layer.

## Discussion

The purpose of this study was to investigate whether applying a sterile transparent film wound dressing would occlude the SO_2_% value collected by the MoorVMS-OXY and it has been proved that it does, but not significantly. However, the application of a double layer of Opsite Flexigrid does significantly alter the results obtained. It also endeavored to consider how this may assist in its clinical application when considering preventing cross infection and / or skin stripping, on the most common weight-bearing ulceration site of the foot [24, 25].

The MoorVMS-OXY has been used in a range of studies proving it is a functional and reliable device to use. It has been used on the following; Wistar rats [26], Sprague-Dawley rats [27], mice [28], pigs [29], and humans; on the forearm [30] and forehead [31]. It is not limited to measuring specific body parts and has the potential to be utilized in clinical practice more frequently than it is at present. This device has been validated as reliable and accurate in measuring SO_2_% in a skin phantom and human skin [32]. Alongside this, results have shown that measuring oxygen and tissue perfusion can be an early indicator of compromised tissue function [30]. Otherwise, there is limited literature on the use of the Moor VMS-OXY and wound healing, with the majority of the previous research focusing on comparing oxygen measurement using this device and others, such as, Near infra-red Spectroscopy (NIRS), pulse oximetry and TcPO_2_. The human studies identified were to; predict results for wound healing with Hyperbaric Oxygen Therapy [33], detect stages of chronic arterial insufficiency of lower extremities [34], and to determine lower limb oxygenation values [35]. These studies each have different limitations, ranging from the participants, measurement sites and comparison devices used. For example, to measure capillary oxygenation using TcPO_2_, the transducer needs to heat to 42 °C, which is not suitable for long-term monitoring and is invasive [33]. NIRS cannot measure oxygen values through adipose tissue, thus limiting its use, [35] and pulse oximetry can only measure capillary SO_2_% on the peripheries; limiting its clinical use [34].

Of these studies, only one [33] mentions using methods to prevent cross-infection occurring which were; sterilizing or sanitizing the device, the participants skin, or using a sterile barrier.

This study illustrated that a double layer of Opsite Flexigrid decreases SO_2_% values and this is likely due to the thickness of Opsite Flexigrid itself and the probe being able to only measure up to a depth of 1-2 mm [8].

When considering the use of a sterile barrier to protect the wound bed, there are many sterile transparent film dressings on the market other than Opsite Flexigrid. Each of these film dressings may have different thicknesses, therefore, the authors recommend trying to identify the thinnest film dressing on the market to establish if this affects the oxygen saturation values obtained.

Currently the results indicate that a double layer of Opsite Flexigrid cannot be used when measuring SO_2_% in wounds because it occludes the probe and affects the values obtained. Using a single layer of Opsite Flexigrid marginally decreases the SO_2_% value compare to no Opsite Flexigrid, however this was shown to have no statistical significant difference, suggesting a single layer of Opsite Flexigrid could be used in clinical practice. It would be beneficial if the study were repeated with a larger sample size to consolidate the findings and provide results more representative to the larger population.

It is acknowledged that skin stripping could occur using a single Opsite Flexigrid, if placed directly on the skin, when measuring SO_2_% in wounds. Therefore, a future study using a single Opsite Flexigrid with the adhesive side stuck to the probe, compared to the single Opsite Flexigrid being stuck on the patient would provide evidence on whether there is a difference in readings or not. This would in turn prevent skin stripping and/or cross infection occuring in patients.

There are limitations concerning the design of this study. Firstly, the relatively small sample size (*n* = 29) alongside the participant gender ratio (5 male: 24 female). A larger sample size generated by a power calculation would enable greater power to detect and extrapolate significant differences (such as gender and ethical diversities), ensuring the study to be more robust. Within the larger sample size, an equal gender ratio would be beneficial because the results would be more representative of the population. We also acknowledge that with within subjects study design there is a potential for carry over effects to be observed. Therefore we recommend readers consider these potential limitations. To potentially help to address these issues, it would be worth randomizing the order of the 3 measurement sets, should the project be repeated to expand the data set.

Strong skin pigmentation can cause compromised results [19]. Using the Fitzpatrick Scale [18] allowed elimination of the participants which had a higher skin score. However, limiting the study participants to skin types 1 and 2. This does not allow a true clinical representation of SO_2_% amongst the entire population and its use in participants with a higher Fitzpatrick Skin score should be avoided. However, if measuring SO_2_% at a wound bed, there is no Melanin present at that point, therefore, further research is required into whether this device can be used in higher Fitzpatrick skin scores if measuring the base of open ulcerations. This would expand its use further in clinic and make it a more versatile machine.

A limitation of the study was that participants were in a rested seated position, rather than laying supine or prone. Being supine or prone may have given more accurate results because it allows the effects of gravity to be eliminated at the foot / probe interface [36, 37]. Previous studies implemented this in their methods so it was not considered a confounding variable [30, 31, 33–35, 37].

## Conclusions

To conclude, there are very few MoorVMS-OXY studies done on humans, but from the literature found, it is used widely showing it is reliable and clinically applicable [26–32]. Oxygen is vital to ensure wound healing, and a lack of oxygen can delay the healing process [2]. Monitoring SO_2_% in patients with ulcers, whom may have PAD and diabetes mellitus, [1] is very important because SO_2_% can be used as a predictor for healing times [2, 6, 9], consequently allowing health professionals to form management plans for each patient. Continuous monitoring of SO_2_% provides tracking of progression and/or regression of wound healing.

From this study’s findings, the application of double Opsite Flexigrid significantly decreases the SO_2_% values obtained compared to when no Opsite Flexigrid is applied, therefore occluding readings generated by the MoorVMS-OXY. This suggests that double Opsite Flexigrid cannot be used when measuring SO_2_%. However, a single Opsite Flexigrid marginally decreases the SO_2_%, but still partially occludes the readings. It is recommended that a single layer could be used, when obtaining measurements from the wound bed, in order to allow easier cleaning of the probe and to minimise cross infection risk. The single layer of Opsite Flexigrid can be applied directly to the probe or to the skin, however the clinician needs to be aware of potential skin stripping, if used on the skin. The results obtained with a single Opsite Flexigrid layer will impact on the results minutely, but not significantly, so should provide some reassurance that the values are as close to those obtained with no Opsite Flexigrid, but while providing greater infection control measures.

## Data Availability

Data is available on request to the Corresponding Author.

## Abbreviations

PAD: Peripheral Arterial Disease
TcPO_2_: Transcutaneous oximetry
NIRS: Near-infrared spectroscopy
SO_2_%: Oxygen saturation percentage
MTPJ: Metatarsophalangeal joint
SD: Standard deviation
